# Effect of Cu Addition on the Corrosion and Antifouling Properties of PEO Coated Zinc-Aluminized Steel

**DOI:** 10.3390/ma15227895

**Published:** 2022-11-08

**Authors:** Luca Pezzato, Alessio Giorgio Settimi, Daniel Fanchin, Emanuela Moschin, Isabella Moro, Manuele Dabalà

**Affiliations:** 1 Department of Industrial Engineering, University of Padova, Via Marzolo 9, 35131 Padova, Italy; 2 Department of Biology, University of Padova, Via U. Bassi, 58/B, 35121 Padova, Italy

**Keywords:** Galvalume, Plasma Electrolytic Oxidation, antifouling, anti-corrosion coatings

## Abstract

In the present work, Plasma Electrolytic Oxidation (PEO) coatings were produced on zinc-aluminized carbon steels (Galvalume commercial treatment). In addition, copper particles of various sizes were introduced into the coating in order to produce samples with antifouling properties. The particles were successfully embedded into the coating. A higher number of embedded particles was observed when these are in sub-micrometric size and obtained in pulsed current. The presence of particles produces significant antifouling properties on the sample’s surfaces during the first 20 days of immersion. The presence of the particles reduces the corrosion resistance in comparison to the samples PEO coated without the particles; however, the corrosion resistance remain higher than the one of the untreated sample.

## 1. Introduction

Steel is one of the most used materials in marine applications, especially for large ships, where carbon steels are often employed. However, this material is subjected to corrosion problems and the substitution with stainless steel is not always possible due to economic or technological problems. Surface treatments on steels and in detail the production of oxide ceramic coatings for corrosion protection is the largely diffused way to improve their corrosion resistance. Between the various surface treatments that aims to produce protective oxide coatings on the surface of metals, PEO seems one of the most promising [1,2].

PEO of metals is a complex electrochemical process, developed from traditional anodizing, that works with higher voltages and current densities [3] in comparison with traditional processes [4]. Due to the high voltage (that has to be above the dielectric breakdown potential of the oxide layer), anodic micro-discharges are formed over the processed surface and produce the growth of an oxide ceramic coating.

PEO coating on aluminum [5], titanium [6] and magnesium alloys [7] has been widely studied and many results are reported in literature, in particular regarding the improvement of the corrosion and wear behavior of the samples [8]. The characteristics of the coatings depends on several process parameters, in particular by the current mode [9] and the current literature showed that coatings produced with pulsed current mode are characterized by improved properties in comparison with the ones produced in direct current [10]. 

Compared to the PEO coating on light alloys, there are relatively fewer studies regarding these coatings produced on steels [11,12,13,14]. Moreover, the quality of PEO coatings obtained on steels is often reduced if compared with the ones obtained on light alloys. One of the possibilities studied in literature to produce PEO coatings on ferrous metals is to perform, before the PEO, a pre-treatment with valve metals. In particular, some works can be found regarding the realization of PEO coatings on aluminized steels [15,16,17]. The main problem regarding this approach is the fact the aluminization of steels is not a so common treatment. Zinc–Aluminum coating (ZA) is instead quite common in steel production, and provides excellent corrosion protection and forming properties compared to traditional zinc layers. Moreover, ZA coating is more common and easy to obtain compared to aluminum coatings.

In the literature are reported some studies regarding the production and characterization of PEO coatings on pure zinc [18,19]. The quality of the obtained coatings was not good and their corrosion protection was quite low. In particular, it was found that despite the thickness of the obtained coatings, the ZnO layers are only slightly protective, due to their semiconducting properties and the presence of cracks and porosities. Better results were found for ZA alloys in different studies published by Pezzato et al. [20], Bian et al. [21] and Guangyin Li et al. [22]. In all the cases was found that the PEO method can produce continuous and dense coating on the ZA27 alloy using silicate, aluminate, and aluminate/borate electrolytes.

The idea of the present work is to work with a very high aluminum content in the ZA layer using a commercial Galvalume coated carbon steel (with 55% Al and 45% Zn in the coatings). This is due to the fact that an increase in the aluminum should increase the quality of the obtained PEO coating. Moreover, Galvalume coated carbon steel was never employed in literature as substrate for Plasma Electrolytic Oxidation coatings.

One of the other characteristics of PEO coating, thanks to the formation of discharge channels, and consequently of a porous surface, is the fact that it also offers a wide range of possibilities to further functionalize the coated surface thanks to the addition of specific additives into the electrolyte [23]. In particular, considering possible marine applications, the possibility of incorporating copper particles into the coatings obtained on aluminum alloys, in order to give to the samples antifouling properties, was already studied by the authors of [24,25]. However, to the best of our knowledge, no works in the literature report the production of antifouling PEO coatings on steels, even if this is of high technological importance, considering that steels, and in particular, carbon steels, are more often used in comparison to aluminum in the production of large ships that work in marine environment.

In this work the possibility to incorporate copper particles into PEO coatings produced on carbon steel, pre-treated with Galvalume treatment, in order to study a possible application in marine environment, was studied. The main goal of the work was to produce on carbon steel a copper-containing PEO coating characterized by good corrosion resistance and antifouling properties that can be employed in marine applications, such as ships. In particular, the idea is to produce an antifouling ceramic coating that can be employed as possible pre-treatment for commercial painting, in order to increase the adhesion of the painting and to assure anticorrosion and antifouling properties in the presence of scratches or defects in the paintings. The microstructure of the coatings and the presence of the particles was studied through SEM and XRD analysis. Corrosion properties were evaluated trough electrochemical tests and trough in situ immersion tests in circulating seawater. After the immersion in circulating sea water, the colonization of the samples after different immersion times in order to estimate the antifouling properties of the copper-containing coatings and to compare them with the ones of the untreated sample and with the ones of the sample PEO coated without copper, was also evaluated. The presence of the copper particles produces a significant antifouling effect during the first 20 days of immersion and slightly reduces the corrosion properties of the coatings that, however, remain higher in comparison to the ones of the untreated sample.

## 2. Materials and Methods

**Figure 1:**
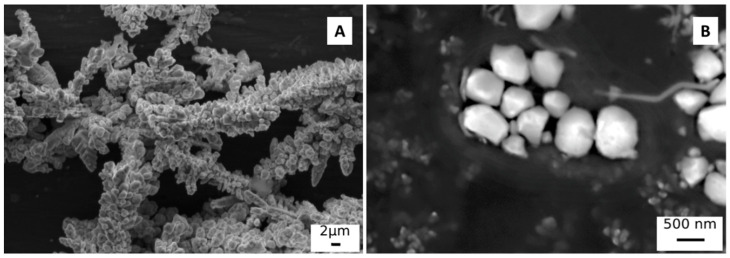
SEM image of the “large” copper particles (**A**) and of the “small” copper particles (**B**).

Commercial Galvalume coated carbon steel samples (55% Al, 45% Zn) were used as substrate for PEO coatings. The samples were previously degreased by ultrasonic bath in acetone for 10 min. The electrolyte used was an aqueous solution of 25 g/L of Na_2_SiO_3_ and 2.5 g/L of NaOH with the addition of 15 g/L of metallic copper particles (all chemicals from Sigma Aldrich, St. Louis, MO, USA). Two different particle sizes were tested; in particular, the particles defined as “large” are characterized by an average size of about 1 µm and the “small” particles by an average size of about 0.45 µm. A SEM micrograph of the different particles can be found in Figure 1 (Figure 1A “large” particles, Figure 1B “small” particles). The dispersion of both “large” and “small” particles in the electrolyte was good and was maintained thanks to continuous magnetic stirring of the electrolyte.

The DC generator used for the PEO coating is a TDK Lambda power supply (TDK-Lambda, Achern, Germany) capable of delivering 2400 W (315 V, 8 A). During the treatment the sample worked as an anode while the cathode was made up of a carbon steel cage. The treatments were performed in galvanostatic mode. Two different operating modes were tested. (a) Direct Current (DC) mode: Current density 0.5 A/cm^2^ in direct current. (b) Unipolar Pulsed Current mode (UPC): 1 A/cm^2^ in pulsed current (with a frequency of 20 Hz and a duty cycle of 50%). Higher current density was employed in the samples obtained in UPC mode due to compensation of the off-time present in pulsed mode, in order to obtain coatings with similar thickness.

The samples produced in direct current modes were treated for 90 s while those obtained in pulsed current for 120 s. The differences in the current density and in the treatment time between the two different modes were chosen on the base of preliminarily tests and of previous works in order to obtain coatings with similar thickness.

After treatment, the samples were washed with deionized water and ethanol and dried with compressed air. The surfaces of the various samples obtained were observed using a Zeiss Stemi 2000-C stereo microscope (Carl Zeiss, Jena, Germany). The cross-sections of the treated samples were obtained by cutting the sample and mounting in epoxy resin, then polished using standard metallographic technique (grinding with SiC abrasive papers from 500 to 1200 grit and polishing with clothes with 6- and 1-micron diamond suspensions). Both the surfaces and the cross sections were analyzed with a SEM Cambridge Stereoscan LEO 440 scanning electron microscope (Leica Microsystem S.r.l., Milan, Italy), equipped with Philips PV9800 EDS microanalysis (Leica Microsystem S.r.l., Milan, Italy), in order to evaluate the morphological characteristics, the thickness of the coating and the elemental composition, as well as clear presence, or not, of the particles. Moreover, EDS elemental maps and line scan analysis were recorded in order to identify elemental distribution into the coatings. The composition of the phases constituting the coating was evaluated by X-ray diffraction analysis (XRD) using a Siemens D500 diffractometer (Siemens, Munich, Germany) using Cu-Kα radiation (step size 0.05°, counting time 5 s).

Corrosion properties of the various samples were evaluated with potentiodynamic polarization (PDP) and electrochemical impedance spectroscopy (EIS) tests at room temperature. PDP tests were performed in a solution containing 0.1 M Na_2_SO_4_ and 0.05 M NaCl, in order to simulate a moderate aggressive environment, with an AMEL 2549 potentiostat (Amel Electrochemistry S.r.l., Milan, Italy), using a saturated calomel electrode as the reference electrode (SCE) and a platinum electrode as the counter electrode with a scan rate of 0.5 mV s^−1^. The EIS measurements were carried out in the previous described solution and electrochemical cell at the value of the open circuit potential and in a frequency range between 10^5^ Hz and 10^−2^ Hz with a perturbation amplitude of 10 mV. The impedance measurements were recorded with a Materials Instrument Spectrometer (Amel Electrochemistry S.r.l., Milan, Italy) coupled with the 2549 Potentiostat and the Z-View software (version 3.3) was used for the fitting of impedance spectra. Both PDP and EIS tests were performed using and exposed area of 1 cm^2^.

To test the antifouling properties, four sets of untreated and treated samples were immersed in triplicate in tanks with circulating seawater at the Hydrobiological Station Umberto D’Ancona in Chioggia (University of Padua).

After the collection, samples were immediately preserved by adding an amount of filtered seawater with 4% formalin, neutralized with hexamethylenetetramine. The biological coverage of the sample surfaces was evaluated after 10, 20, 30 and 40 days of immersion with visual observation with a Zeiss Stemi 2000-C stereomicroscope (Carl Zeiss, Jena, Germany). For the determination of the microphytobenthos community structure, the surface of each sample was scraped and the microorganisms were observed with an inverted Leitz Diavert microscope (Leitz, Stuttgart, Germany), equipped with phase contrast. The scraped biofilms were distributed in a settling chamber and the microalgae analyzed from 60 randomly selected fields of view with a magnification of 400×. Microalgae were identified using standard keys, in particular for diatoms [26,27,28] were used as reference.

Moreover, to identify the microphytobenthos taxa present in the biofilms a series of samples was analyzed through SEM Cambridge Stereoscan LEO 440, after proper gold sputtering.

## 3. Results and Discussion

### 3.1. Production and Characterization of PEO Coatings

**Figure 2:**
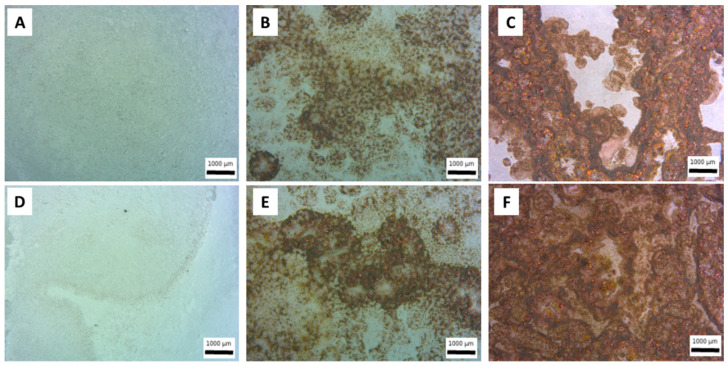
Stereo microscope images of the surfaces of PEO coatings obtained working at 0.5 A/cm^2^ in DC mode without particles (**A**), with large particles (**B**) and with small particles (**C**) and working at 1 A/cm^2^ in UPC without particles (**D**), with large particles (**E**) and with small particles (**F**).

Metallic copper particles were inserted into PEO coatings, produced on aluminum-galvanized steel, in order to functionalize the surfaces and confer antifouling properties. The insertion of the particles into the coatings took place by simply adding them to the electrolyte used for the process. In order to verify the presence of particles inside the coating and qualitatively assess the number of particles incorporated, the various samples were observed under optical and electronic microscope. The results of the observation of the surfaces under the stereo microscope can be seen in Figure 2, in order to understand the global microstructure of the coating at low magnification. The presence of copper particles, which are then successfully incorporated into PEO coatings, is evident both in the samples obtained with large particles (Figure 2B,E) and in those obtained with small particles (Figure 2C,F). An evident correlation can also be observed between the particle size and the amount of Cu incorporated, with small particles that are more easily incorporated within the coating (compare regarding this Figure 2C with Figure 2B and Figure 2F with Figure 2E). This fact can be linked with the dimension of the pores; the particles that have a smaller size than the pores are in fact more easily incorporated into the coating. This, according to Lu et al. [23], showed that pores on the coating surface can be considered as uptake paths for particles.

The method of application of the current also influences the quantity of particles that are incorporated into the coating; in fact, in pulsed current mode (Figure 2E,F), a greater quantity of particles incorporated into the coating can be observed in comparison to DC mode (Figure 2B,C). This fact can be noted both in the case of large particles (Figure 2E in comparison with Figure 2B) and in the case of small particles (Figure 2F in comparison with Figure 2C) and is also in accordance with literature [29].

**Figure 3:**
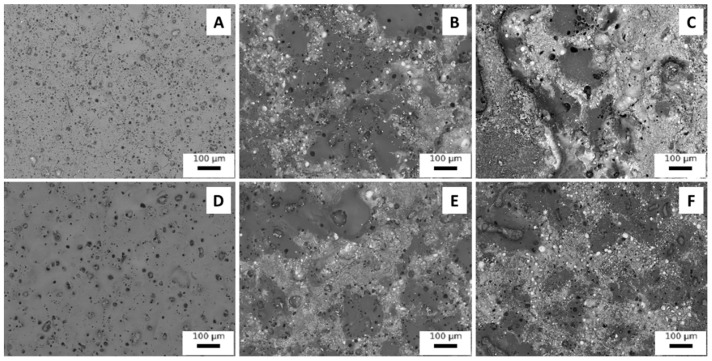
SEM images of the surfaces of PEO coatings obtained working at 0.5 A/cm^2^ in DC mode without particles (**A**), with large particles (**B**) and with small particles (**C**), and working at 1 A/cm^2^ in UPC without particles (**D**), with large particles (**E**) and with small particles (**F**).

**Figure 4:**
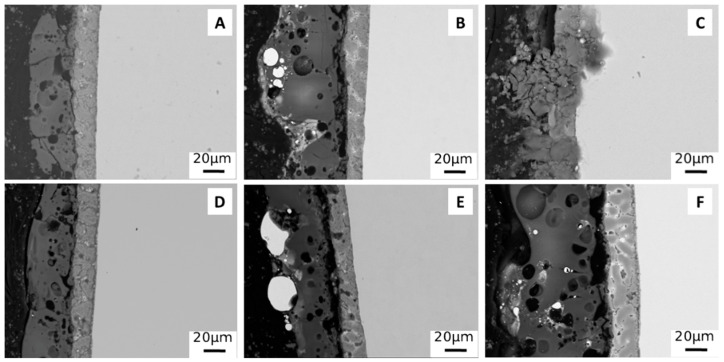
SEM images of the cross section of PEO coatings obtained working at 0.5 A/cm^2^ in DC mode without particles (**A**), with large particles (**B**) and with small particles (**C**), and working at 1 A/cm^2^ in UPC without particles (**D**), with large particles (**E**) and with small particles (**F**).

In order to study the coatings obtained in more detail, SEM observations of the sample were also carried out and the results are shown in Figure 3 for the surfaces and in Figure 4 for the cross sections. Considering the observation of the samples’ surfaces, first of all, the typical porous morphology of PEO coatings in Figure 3A (average pore size 10 microns) and 3D (average pore size 30 microns) can be observed, and it can be also noted that the UP mode produces a denser PEO layer. The consideration performed on the quantity of incorporated particles can be clearly confirmed and a mixed mechanism of inert and reactive incorporation can be observed. Part of the particles are, in fact, inertly incorporated and maintain their shape, whereas another part of the particles is melted and re-solidified due to the extremely high local temperatures that can be reached during PEO process. The melting point of copper is, in fact, remarkably lower than the temperature of arc plasma that, as evidenced by Lee et al. [30], ranges between 1800 and 2370 °C. The melted particles resulted as grey-white zones, without clear shape, on the surface of the coatings (Figure 3B,C,E,F).

Considering the cross section (Figure 4), in all samples, obtained both with and without particles, the presence of both the Galvalume layer and the PEO coating can be observed.

In all samples, the PEO coating has a thickness of about 20 microns, except in the sample obtained with small particles at 0.5 A/cm^2^ in DC mode (Figure 4C). In this case, the PEO coating appears not completely formed, due to the too violent discharge phenomena created by the direct current with the presence of small particles. In the samples produced with particles in the electrolyte, the presence of the particles themselves (white areas) within the PEO coating is observed. These appear as large agglomerates in the case of the use of large particles (Figure 4B,E) while they are small in the case of the use of small particles (Figure 4C,F). The size of the particles significantly affects their distribution mode within the coating. In fact, the large particles (Figure 4B,E) are mainly concentrated on the surface of the coating, while the small particles, in particular working in pulsed current (Figure 4F), are distributed along the entire thickness of the coating. This appears to be in accordance with the literature [31] where it is highlighted how particles of sub micrometric size are able to enter the discharge channels that are formed during the formation of the PEO coating and, consequently, remain trapped inside the ceramic layer. Large particles, on the other hand, tend to simply “decorate” the surface of the coating itself.

**Figure 5:**
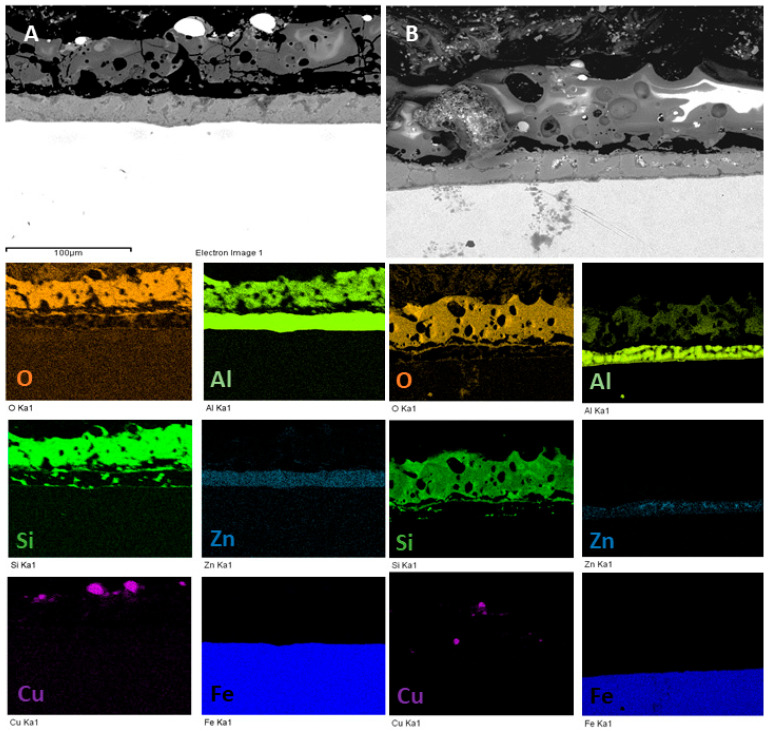
EDS elemental maps of the samples obtained working at 1 A/cm^2^ in UPC with large particles (**A**) and with small particles (**B**).

To confirm these observations and to evaluate the chemical distribution of the elements into the coatings, EDS elemental maps were performed on the most promising samples so the ones obtained with the particles in UPC mode, and the results are reported in Figure 5. The uniform distribution of Si, Al and O and the presence in the Galvalume Sub-layer of Al and Zn can be clearly noted in the PEO coated samples. Considering the copper particles, the previously reported observations can be confirmed: large particles “decorate” only the surface of the coating (Figure 5A), whereas small particles (Figure 5B) are also present inside the coatings, trapped in the discharge channels.

**Figure 6:**
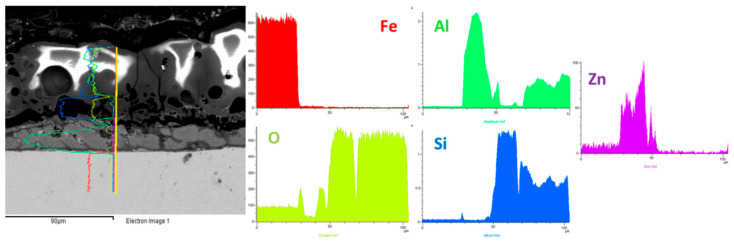
EDS elemental line analysis of the sample obtained working at 1 A/cm^2^ in UPC with small particles.

In order to also evaluate the composition and the thickness of the ZA layer and to study the interface between the ZA layer and the PEO coating, EDS line analysis was performed and the results obtained in the sample produced at 1 A/cm^2^ in UPC with small particles are reported in Figure 6. First of all, it can be noted that the ZA layer is composed almost equally by Zinc and Aluminum and the thickness of the layer is around 20 microns. The resulting PEO layer is instead composed mainly of Al, Si and O with the Zinc not contributing significantly in the formation of the coating. Considering the interface between the ZA layer and the PEO coating, a decrease in the aluminum in comparison with the PEO layer can be observed. Therefore, at the interface, there is a concentration in the silicon content with the aluminum that increase instead in the external part of the coating. This is probably related to the fact that silicon oxidation occurs before that of aluminum, causing an increase in the silicon oxide content at the interface between the ZA layer and the PEO layer.

**Figure 7:**
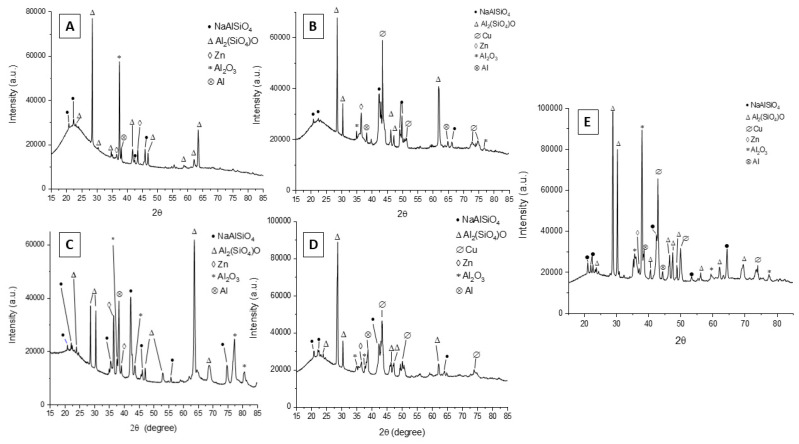
X-ray diffraction patterns of the samples obtained working at 0.5 A/cm^2^ in DC mode without particles (**A**) and with large particles (**B**) and working at 1 A/cm^2^ in UPC without particles (**C**), with large particles (**D**) and with small particles (**E**). Sample obtained at 0.5 A/cm^2^ in DC with small particles not reported due to failure of the coating.

To identify the various phases, present within the coating, XRD analysis was also performed, and the results are reported in Figure 7. From the spectra, it can be seen that the coating consists mainly of aluminum oxide (Al_2_O_3_) and aluminosilicates (Al_2_SiO_5_ and NaAlSiO_4_). There are also clearly present peaks of copper, relative to the particles in the samples obtained with the particles, and those of Al and Zn, relative to the underlying Galvalume coating. The composition of the obtained coating resulted in accordance with the ones of the electrolyte and of the substrate and is also confirmed by results previously obtained in the literature [24]. XRD spectra of the sample obtained at 0.5 A/cm^2^ in DC with small particles are not reported due to failure of the coating. From the comparison between the different samples, no significant differences can be observed in terms of phase composition.

### 3.2. Corrosion Properties

**Figure 8:**
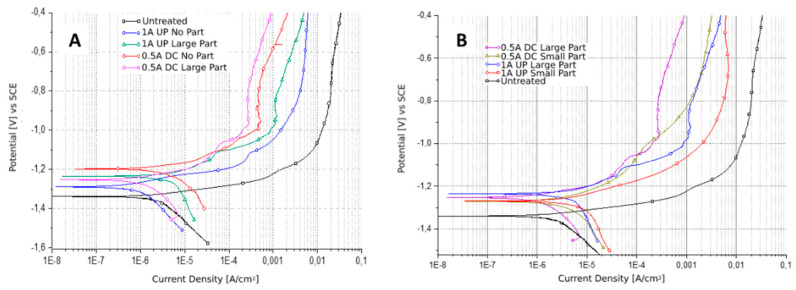
Results of potentiodynamic polarization tests (PDP) performed on the samples produced without particles and with “large” particles (**A**) and of the samples produced with “small” and “large” particles (**B**). Test electrolyte: 0.1 M Na_2_SO_4_ and 0.05 M NaCl.

In order to preliminarily evaluate the effect of the presence of copper particles on the corrosion resistance of the obtained coatings, potentiodynamic polarization tests were carried out on the various samples. The results of the samples obtained with the particles were compared with those of the samples obtained without particles and with those of the untreated sample (steel with Galvalume coating). The results can be found plotted in Figure 8 and the values of corrosion potentials and corrosion current densities, graphically extrapolated, are reported in Table 1. Generally, it can be observed that the addition of the particles leads to a decrease in corrosion resistance (and so to an increase in the corrosion current) compared to samples obtained by PEO treatment without particles, probably due to galvanic contact phenomena between the copper and the underlying metal (with the formation of Cu-Al and Cu-Zn galvanic couples) and to variations in the discharge mechanisms due to the presence of copper in the electrolyte. Samples obtained with small particles show a greater decline in corrosion resistance than those obtained with large particles, probably due to the greater number of particles incorporated.

**Figure 9:**
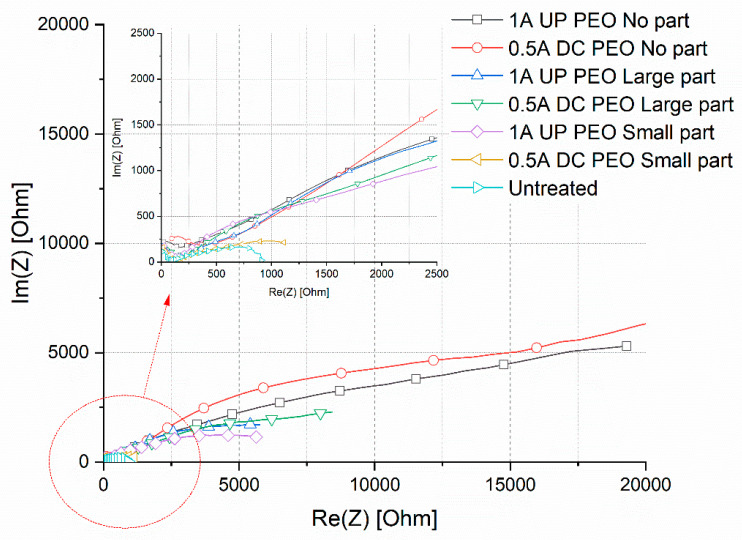
Results of the EIS tests in term of Nyquist plot. In the high left, the zone at the high frequencies can be observed in more detail. Test electrolyte: 0.1 M Na_2_SO_4_ and 0.05 M NaCl.

**Figure 10:**
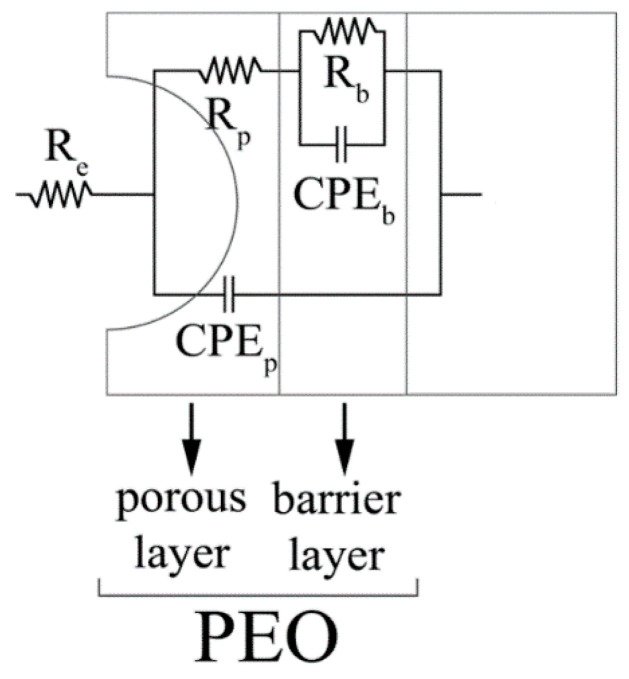
Equivalent circuit employed to fit the experimental data coming from EIS test.

In order to quantitatively evaluate the corrosion performance of the samples and to study the effect of particles addition on the corrosion resistance, EIS tests were also performed in the same electrolyte employed in PDP tests. The results in term of Nyquist plot are reported in Figure 9, where dots represent the experimental data. The data from EIS tests were also fitted using the circuit reported in Figure 10 and the results of the fitting, that are graphically represented in Figure 9 as lines, can be found reported in Table 2.

The equivalent circuit was chosen on the basis of the literature on PEO coatings [32] that suggests to employ a double circuit (Figure 10) to fit data coming from PEO treated samples. In particular, this permits consideration of the presence of an inner and an external layer, called, respectively, barrier layer and porous layer. A good fitting quality was obtained, as confirmed by the low values of chi-squared in Table 2 and by the good correspondence between dots and lines in Figure 9. The untreated sample was instead fitted using a simple Randles circuit in order to consider the presence of the natural oxide layer. Considering the physical meaning of the different elements of the equivalent circuits in Figure 10, R_e_ represents the resistance of the electrolyte, R_p_ and CPE_p_ represents the porous layer of PEO coating, and R_b_ and CPE_b_ the barrier layer. CPEi (Constant Pahse Elements) were used in the equivalent circuits instead of capacitances due to the fact that the measured capacitance is not ideal. The impedance of a CPE has the form:

(1) $$1/Z=Y=Q{}^{\circ}\;{\left(j\;\mathrm{omega}\right)}^{n}$$

where Q° has the numerical value of the admittance (1/Z) at omega = 1 rad/s. When this equation describes a capacitor, Y = C (the capacitance) and the exponent n = 1. For a constant phase element, the exponent n < 1. The “double-layer capacitor” on real cells often behaves like a CPE instead of like a capacitor. The values of Q_i_ and n_i_ in Table 2 refer to the CPE_i_ of the two layers that constitute the PEO coating. The value of Χ^2^ represents the statistical error during the fitting of the experimental data.

In the Nyquist plot reported in Figure 9, the real part of the impedance at low frequencies can be considered as qualitative indication of the corrosion properties of the sample. Considering this, it can be clearly noted that the EIS results confirm the indication given by the PDP tests. First of all, it can be clearly noted that all the PEO treated samples are characterized by improved corrosion properties in comparison with the untreated one. The sample with the lower corrosion properties was, in fact, the one 0.5 DC with small particles, in which the PEO coating was not completely formed, whereas the best corrosion properties are obtained on the samples without the copper particles. All the samples obtained with particles (expect for the one 0.5DC small particles) are characterized by similar corrosion resistance, lower than the one of the samples without particles, due to the galvanic couple caused by the presence of copper. The qualitative indication obtained from the observation of the Nyquist plot is also confirmed by the results of the fitting of the experimental data reported in Table 2. Considering this data, first of all, it can be observed in all the samples, as typical for PEO coatings, that the value of R_B_ is higher than the one of R_P_, due to the increased corrosion properties of the barrier layer. The value of R_B_ for the samples treated without particles (1A UP and 0.5 DC) was around 30,000 ohm cm^2^, whereas the one of the samples treated with the particles was around 10,000 ohm cm^2^, confirming the reduction in the corrosion properties due to the galvanic couple between the copper and the aluminum substrate. Excluding the sample 0.5DC small particles, characterized by a value of R_B_ around 1000 ohm cm^2^ due to incomplete coating formation, the sample with the lower value of R_B_ was the one 1A UP with small particles (9126 ohm cm^2^). This can be linked with the SEM observation where the sample 1A UP with small particles was the one with the higher amount of incorporated particles and so with higher problems of galvanic coupling. In all the PEO treated samples, the polarization resistance was higher than the one of the untreated sample, characterized by the presence of only the natural oxide layer (950 ohm cm^2^).

### 3.3. Antifouling Properties

**Figure 11:**
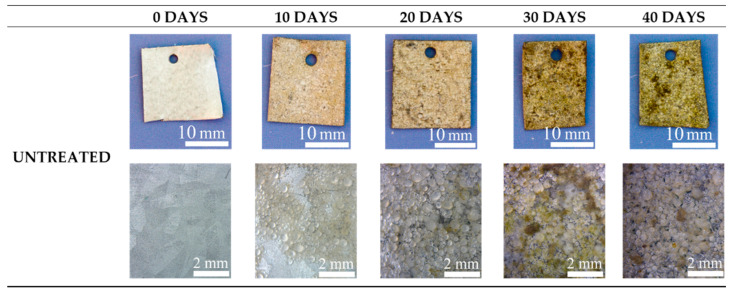
Visual observation (on the upper part) and stereo-microscope observation (on the bottom part) of the untreated samples after 0, 10, 20, 30 and 40 days of immersion in circulating seawater at the Hydrobiological Station Umberto D’Ancona.

**Figure 12:**
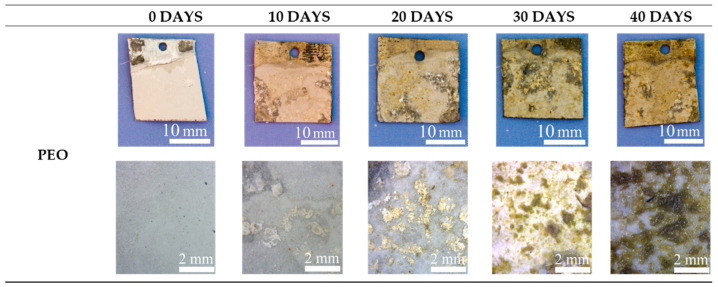
Visual observation (on the upper part) and stereo-microscope observation (on the bottom part) of the PEO treated samples (1A UP No part) samples after 0, 10, 20, 30 and 40 days of immersion in circulating seawater at the Umberto D’Ancona Hydrobiological Station.

**Figure 13:**
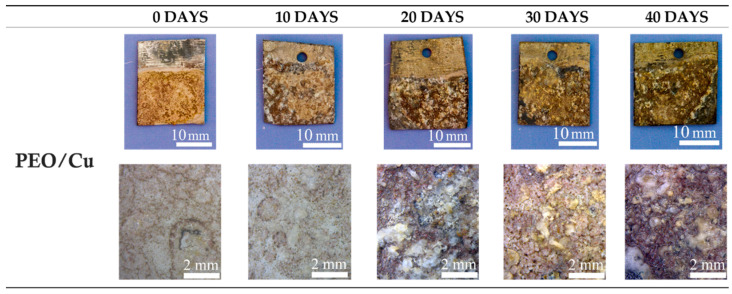
Visual observation (on the upper part) and stereo-microscope observation (on the bottom part) of the PEO treated samples with copper particles (1A UP small part) samples after 0, 10, 20, 30 and 40 days of immersion in circulating seawater at the Umberto D’Ancona hydrobiological station.

The antifouling properties were evaluated by immersing the samples in circulating seawater for 40 days and analyzing the surfaces after each 10 days. In Figure 11, Figure 12 and Figure 13, the visual and the stereo microscope observations of the immersed samples after different immersion times are reported. In particular, in Figure 11, the untreated sample is reported, in Figure 12, the PEO treated sample (1A UP without particles), and in Figure 13, the sample PEO treated with the presence of copper particles (1A UP with small particles). The immersion tests were performed on the sample 1A UP with small particles; from the previously reported observations, this was in fact the one with the higher number of incorporated particles, and so the more promising in terms of antifouling properties. In fact, as reported in the literature [24], the quantity of the antifouling agent plays a key role in the colonization. For comparison reasons, the immersed sample without particles was the corresponding one, 1A UP without particles. Considering the untreated sample (so the one with only the ZA layer, Figure 11), already after 10 days of immersion, a remarkable colonization can be clearly observed and after 40 days almost all the surface resulted covered. Moreover, remarkable corrosion phenomena can be noted with the presence of evident cavities on the surface already after 10 days. The PEO coatings without particles (Figure 12) do not reduce the fouling phenomena that instead were increased, as can be observed by the presence of bigger green zones in the surface after 40 days of immersion. This fact was in accordance with previous work of the authors [25] and is linked with the surface morphology of the PEO layer. As was also observed in the SEM analysis, the surface of PEO coatings is, in fact, porous and in these pores, the algae can grow faster. Instead, no remarkable corrosion phenomena can be observed on the surface, even after 40 days of immersion, thanks to the protection given by the PEO layer. Considering the sample PEO treated with the copper particles (Figure 13), a remarkable antifouling effect of the copper particles during the first 10 and 20 days of immersion can be observed. In fact, after 10 and 20 days, no colonization is observable. After 30 and 40 days, the colonization seems to start also in the sample PEO treated with the copper particles, probably due to the fact that the complete coverage of the surface of the samples by the biofilm significantly reduces the antifouling effect of the copper particles present in the PEO underlayer. Furthermore, the corrosion properties of the samples PEO/Cu were good with no evidence of corrosion phenomena even after 40 days. Considering both these facts, the PEO coating with copper particles can be considered as promising pre-treatment for antifouling paints on steels. In fact, the PEO layer will protect from corrosion in presence of scratches of defects in the varnish and the presence of the copper particles will assure the antifouling properties, at least during the first days of immersion before that proper maintenance can be performed. Moreover, the porous morphology of the PEO surface make it particularly suitable for increasing the mechanical bond between the metal and the organic coating.

### 3.4. Biological Colonization

The observation at the light microscope of the microphytobenthos community of the samples showed a predominance of diatoms, represented by pennate forms, while the centric ones were absent. This was due to their capability to attach and glide on a substrate thanks to the presence of raphe or pores, structures involved in the secretion of extracellular polymeric substances (EPS) [33,34]. In the later phases of the colonization (30 and 40 days), filamentous cyanobacteria were also present, mainly in the PEO treated samples (PEO and PEO/Cu).

**Figure 14:**
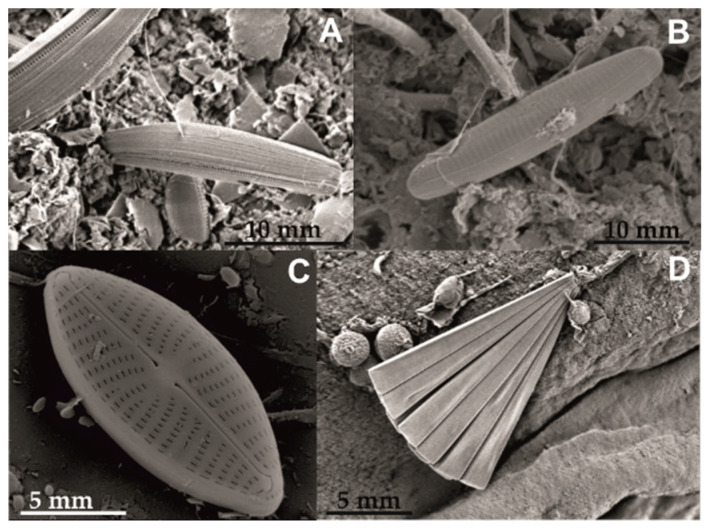
Scanning electron micrographs showing some diatoms found in the samples. (**A**) *Amphora* sp., bar = 10 µm; (**B**) *Navicula* sp., bar = 10 µm; (**C**) *Navicula* sp., bar = 5 µm; (**D**) *Licmophora* sp., bar = 5 µm.

After 10 days, PEO and PEO/Cu treatments showed a lower number of taxa than the control one, suggesting a selective effect of PEO on the adhesion of some taxa (Table 3). All the samples showed erect (attached to surfaces by stalks), adnate (forms closely appressed to the substratum) and motile species, mainly belonging to the genera *Amphora* (Figure 14A), *Cylindrotheca*, *Haslea*, *Navicula* (Figure 14B,C), *Licmophora* (Figure 13D)*, Nitzschia* and *Tabularia*. In the untreated sample, the cell number for each taxon was almost homogeneous, in the PEO and PEO/Cu treated samples, some genera (e.g., for PEO, *Psammodyction panduriforme*, for PEO/Cu *Thalassionema nitzschioides*) were present with a high number of cells.

In the following immersion times (20 and 30 days), while PEO maintained a scarce number of taxa, PEO/Cu showed an increase in the biodiversity, until reaching the same level as the untreated sample. This could be probably due to the roughness of the surfaces, favoring a better adhesion of the benthic microorganisms.

After 40 days of immersion, a high number of taxa was registered in all the samples (control and PEO treatments). The decrease in the antifouling capability in the samples treated with PEO and PEO/Cu showing a number of taxa similar to the control one could be probably due to the complete coverage of the substrate by microalgal biofilm that cancels the effect of the treatments for the colonization by the upper foulers.

## 4. Conclusions

The following conclusion can be summarized:

Copper particles were successfully incorporated into PEO coatings obtained on steels after Galvalume treatment.

The number of incorporated particles increases by reducing the particle size and switching from a direct current to a pulsed current mode.

The presence of particles reduces the corrosion resistance of PEO coatings, which still remains higher than that of steel after Galvalume treatment.

The presence of the copper particles produces a significant antifouling effect during the first 20 days of immersion, whereas for increased immersion times, the presence of copper particles does not show effect in reducing the colonization phenomena.

PEO treatment with copper particles can be considered as promising pre-treatment for painting steel due to the fact that assure corrosion and antifouling properties even in presence of defect or scratches in the varnish, at least during the first 20 days of immersion before that proper maintenance can be performed.

## Figures and Tables

**Table 1:** Corrosion current densities and corrosion potentials graphically extrapolated from the potentiodynamic polarization plots.

Sample	E_corr_ (V)	I_corr_ (A/cm^2^)
Untreated	−1.34	2.04 × 10^−6^
0.5 DC No part	−1.19	2.28 × 10^−6^
0.5 DC large part	−1.25	7.83 × 10^−7^
0.5 DC small part	−1.27	2.07 × 10^−6^
1A UP No part	−1.28	5.17 × 10^−7^
1A UP large part	−1.23	2.46 × 10^−6^
1A UP small part	−1.26	3.22 × 10^−6^

**Table 2:** Results of the fitting of the experimental data coming from EIS tests.

Sample	R_e_ (Ωcm^2^)	R_P_ (Ωcm^2^)	R_B_ (Ωcm^2^)	Q_P_ (F cm^−2^Hz^1−n^)	n_P_	Q_B_ (F cm^−2^Hz^1−n^)	n_B_	Χ^2^
Untreated	20	-	950	-	-	3.8 × 10^−5^	0.8	0.0001
0.5 DC No part	21	442	32,129	2.97 × 10^−5^	0.51	1.22 × 10^−7^	0.7	0.0003
0.5 DC large part	20	432	12,351	1.57 × 10^−5^	0.84	1.3 × 10^−5^	0.62	0.0008
0.5 DC small part	17	83	1148	8.2 × 10^−4^	0.75	1.7 × 10^−7^	0.75	0.0004
1A UP No part	22	200	32,172	5.51 × 10^−5^	0.65	5.93 × 10^−6^	0.8	0.0006
1A UP large part	20	250	13,145	3.1 × 10^−4^	0.62	1.49 × 10^−6^	0.71	0.0004
1A UP small part	16	448	9126	3.06 × 10^−6^	0.84	1.8 × 10^−5^	0.68	0.0001

**Table 3:** Number of taxa registered on the different samples during the experimental period.

	10 Days	20 Days	30 Days	40 Days
Untreated	21	19	23	21
PEO	11	17	16	20
PEO/Cu	14	16	22	22

## Data Availability

The raw/processed data required to reproduce these findings cannot be shared at this time as the data also form part of an ongoing study.
